# Invisible invaders: unveiling the carcinogenic threat of microplastics and nanoplastics in colorectal cancer-a systematic review

**DOI:** 10.3389/fpubh.2025.1653245

**Published:** 2025-08-19

**Authors:** Junkai Wen, Yuhua Lin

**Affiliations:** ^1^Department of Oncology, Shuguang Hospital, Shanghai University of Traditional Chinese Medicine, Shanghai, China; ^2^Department of Respiratory Medicine, Xiamen TCM Hospital Affiliated to Fujian University of Traditional Chinese Medicine, Xiamen, China; ^3^Department of Respiratory Medicine, Xiamen Hospital, Dongzhimen Hospital, Beijing University of Chinese Medicine, Xiamen, China

**Keywords:** carcinogenic mechanisms, gut barrier disruption, DNA damage, oxidative stress, environmental toxicology, human exposure

## Abstract

**Objective:**

Microplastics (MPs, 0.1–5000 μm) and nanoplastics (NPs, 0.001–0.1 μm) are ubiquitous environmental pollutants with strong persistence and bioaccumulation, posing significant threats to human health. Given their ability to penetrate biological barriers, accumulate in the food chain, and infiltrate human gastrointestinal tissues, humans ingest and inhale over 70,000 microplastic particles annually, and the observed higher abundance of MPs/NPs in colorectal tumor tissues in epidemiological data, this systematic review aims to explore the link between MPs/NPs exposure and colorectal cancer (CRC) carcinogenesis.

**Methods:**

This study synthesizes 20 years of relevant research to systematically analyze the association between MPs/NPs exposure and CRC development.

**Results:**

Key findings reveal that MPs/NPs enter the body via ingestion, inhalation, and dermal contact, translocating across biological barriers to induce DNA damage and oxidative stress through reactive oxygen species overproduction. They disrupt intestinal barrier function by reducing tight junction proteins, trigger chronic inflammation via pro-inflammatory cytokines, and cause gut microbiota dysbiosis. Additionally, MPs/NPs act as “Trojan horses”, adsorbing toxicants (e.g., bisphenol A) and pathogens, which exacerbate cytotoxicity and activate carcinogenic pathways.

**Conclusion:**

This review highlights the potential carcinogenic risk of MPs/NPs in CRC, deepens understanding of their mechanistic roles in carcinogenesis, and provides insights for the scientific management of MPs/NPs pollution.

## 1 Introduction

Plastic, valued for its low cost, wide applications, and stable properties, has rapidly expanded globally, with production rising annually (1, 2). Unlike initial beliefs of inertness, its high resistance to degradation leads to ecosystem accumulation due to limited recycling, posing a major pollution threat. Through physical, chemical, and biological processes, plastic breaks down into microplastics (MPs) and nanoplastics (NPs)—tiny plastic particles with diameters ranging from 0.1 to 5,000 μm are referred to as MPs, while those with diameters between 0.001 and 0.1 μm are called NPs (3, 4).

**Figure 1:**
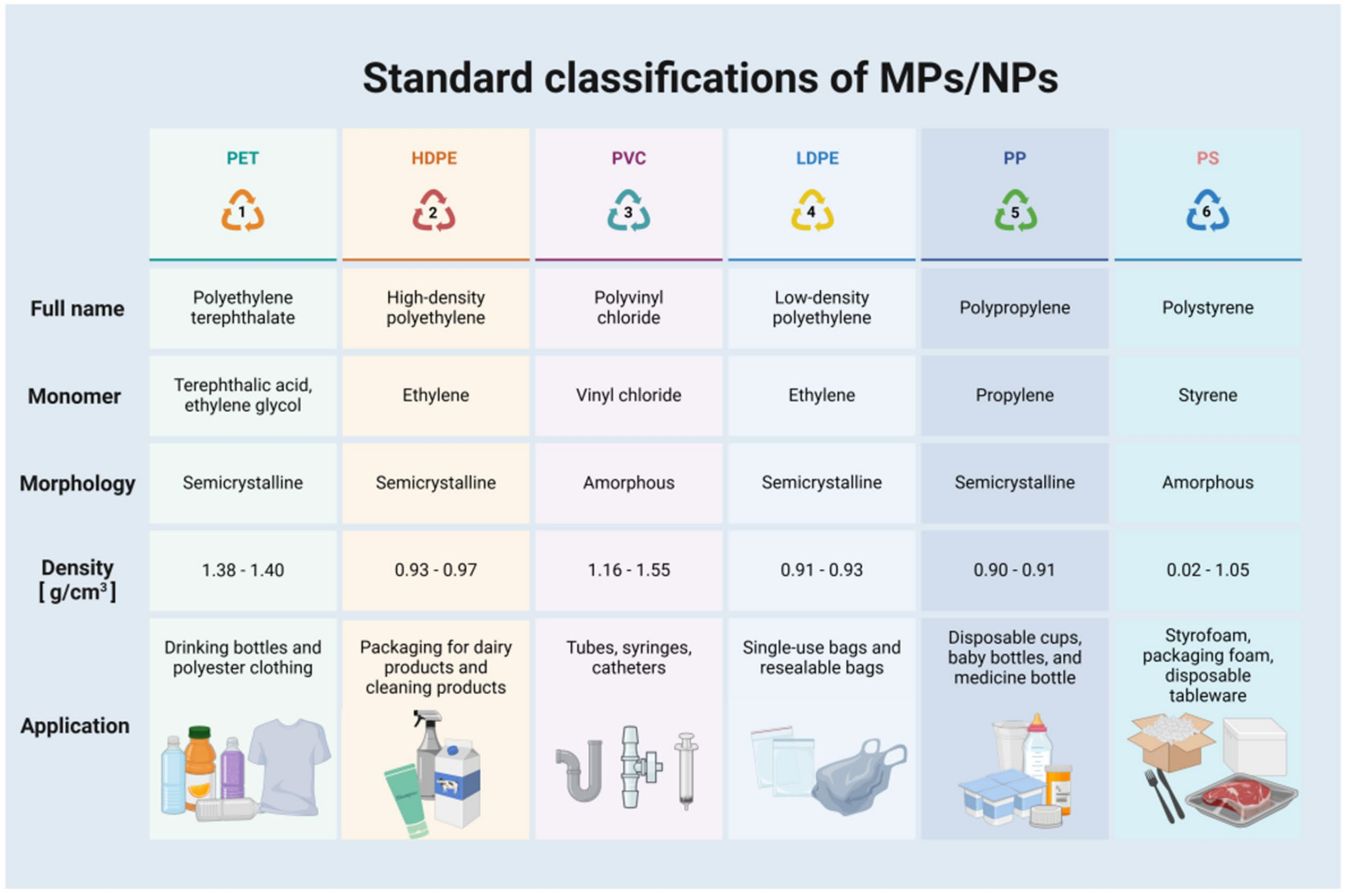
Standard classifications of MPs/NPs. Created with BioRender.com.

MPs are categorized into primary and secondary types based on origin: primary MPs are industrial plastic particles or microbeads manufactured for specific uses, whereas secondary MPs form through environmental degradation (e.g., photodegradation, abrasion, and water erosion) of larger plastic items, presenting as fibers, granules, fragments, or films (5). Due to their complex nature as aggregates of various-sized sheets, fragments, and fibers, MPs/NPs in the environment are further classified by chemical structure and source into major types including polyethylene terephthalate (PET), high-density polyethylene (HDPE), low-density polyethylene (LDPE), polyvinyl chloride (PVC), polypropylene (PP), and polystyrene (PS) (6–8) (Figure 1).

**Figure 2:**
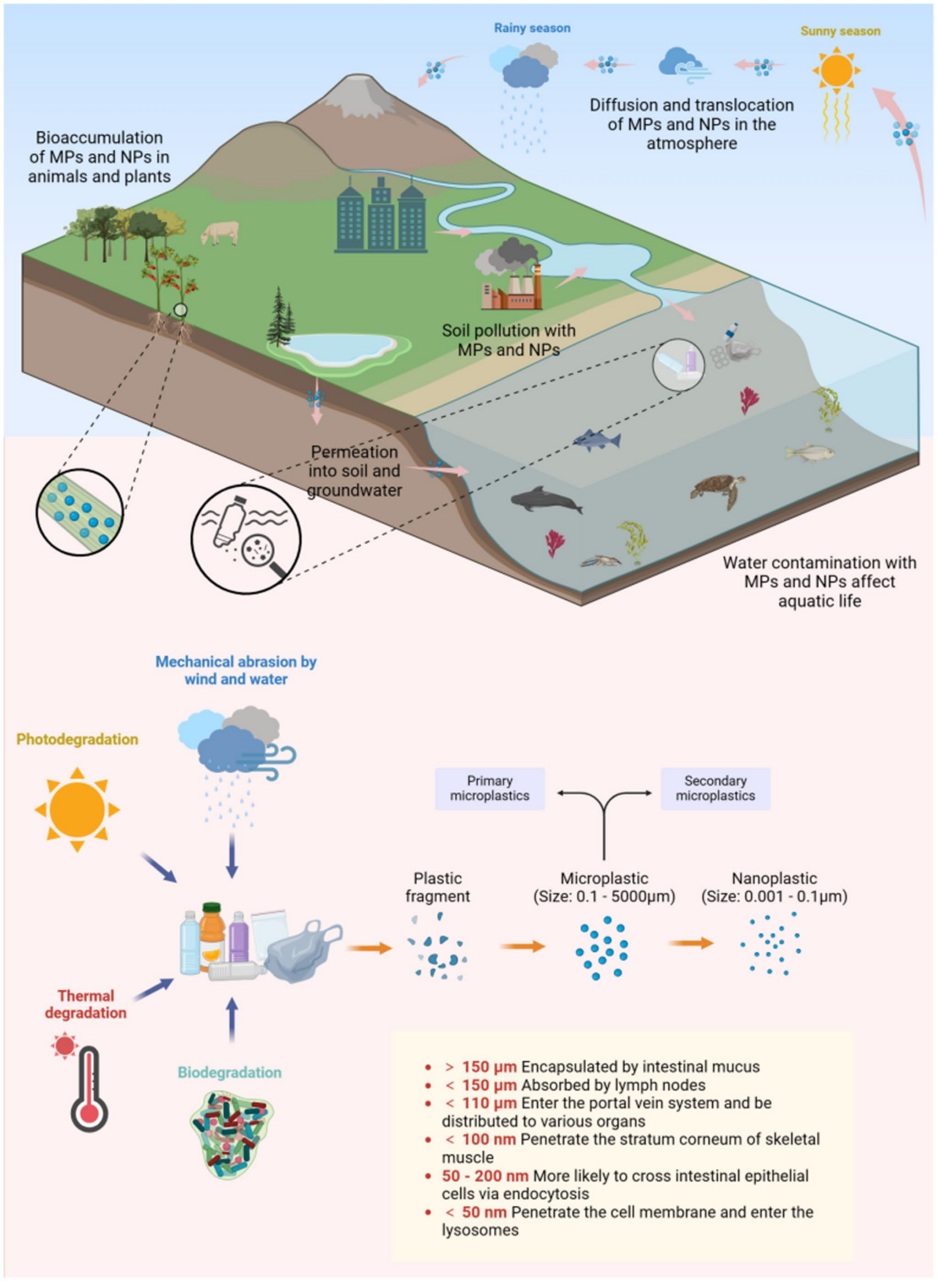
The transfer, dispersion, degradation, and internalization of MPs/NPs. Created with BioRender.com.

These particles (MPs/NPs), transported by ocean currents and winds, are ingested by organisms (Figure 2). In adult zebrafish, MPs/NPs cause gut microbiota imbalances, abnormal intestinal cell division, and wall fissures (9), and after 21 days, dysbiosis impacts metabolism (10). Moreover, MPs/NPs accumulate in organisms, move up the food chain, and have entered the human food chain (Supplementary Figure 1), being detected in drinking water, foods, placenta, and colorectal cancer (CRC) samples (11, 12) (Table 1). Estimates suggest humans ingest and inhale over 70,000 microplastic particles yearly (13), and the global MP quantity may increase fiftyfold by the end of this century (14), making their health impact a key research topic.

**Table 1:** Accumulation of MPs/NPs in the human body.

Sample type (tissue/organ)	Shape	Size	Abundance	Composition	Detection methods	Reference
Colon tissue	Filaments and fibers	(1.1 ± 0.3) mm	(28 ± 15) n·g^−1^	PC, PA, and PP	FTIR and SM	(7)
Colon tissue	Not mentioned	1 ~ 1,299 μm	(207 ± 154) n·g^−1^	PE, PMMA, and PA	ATR-FTIR, RS, and OM	(15)
Colorectal adenocarcinoma tissue	Not mentioned	1 ~ 613 μm	(702 ± 504) n·g^−1^	PE, PMMA, and PA	ATR-FTIR, RS, and OM	(15)
Normal colorectal tissue adjacent to adenocarcinoma	Not mentioned	1 ~ 743 μm	(207 ± 154) n·g^−1^	PE, PMMA, and PA	ATR-FTIR, RS, and OM	(15)
Cirrhotic liver tissue	Not mentioned	4 ~ 30 μm	3.2 n·g^−1^	PS, PET, and PVC	FTIR with Nile red staining and RS	(83)
Feces	Fragments, films, spheres, and fibers	50 ~ 500 μm	2 n·g^−1^	PP, PET, PS, and PE	FTIR	(31)
Lung tissue	Microbeads (49%), fragments (43%), and films (8%).	1 ~ 2,500 μm	(0.7 ± 0.8)n·g^−1^	PP and PET	μFTIR	(84)
Blood	Not mentioned	≥ 700 nm	1.6 μg·ml^−1^	PET, PE, PS, and PMMA	Py-GC/MS	(85)
Placental tissue	Fragments, fibers, and films	20 ~ 307 μm	(2.7 ± 2.6) n·g^−1^	PVC (43%), PP (15%), and PBS (11%)	LDI	(86)
Breast milk	Fragments (91%) and microspheres (9%)	≤ 3 μm (29%), 4 ~ 9 μm (24%), ≥ 10 μm (47%)	Not mentioned	PE (38%), PVC (21%), and PP (17%)	SM and RS	(87)

PC, Polycarbonate; PA, Polyamide; PE, Polyethylene; PMMA, Polymethyl methacrylate. ATR-FTIR, Attenuated total reflectance-Fourier transform infrared spectroscopy; SM, Stereo microscope; RS, Raman spectroscopy; OM, Optical microscope; Py-GC, Pyrolysis-gas chromatography; MS, Mass spectrometry; LDI, Laser direct infrared imaging.

As early as 2009, Erren et al. (15) suggested that plastics might be linked to the rising incidence of cancer in living organisms. In recent years, an increasing number of *in vivo* and *in vitro* experiments have confirmed that MPs/NPs have potential impacts on the gastrointestinal tract (16) and may promote the occurrence and progression of colorectal cancer (CRC) (17–19). The study by Cetin et al. (20) further found that the number of MPs/NPs in normal human colon tissue is significantly lower than that in the colon tumor tissue of rectal adenocarcinoma patients. CRC cell lines also show increased uptake and intracellular accumulation of MPs/NPs.

Notably, MPs/NPs have been ubiquitously detected across diverse environmental matrices, including marine systems such as polar and Mediterranean waters, freshwater bodies like large lakes and subalpine lakes, sediments, the atmosphere, soil, food, and terrestrial/anthropogenic sources such as agricultural waste, aquaculture systems, and landfill leachate—underscoring their extensive dissemination through ecosystems (21). Prior research has predominantly focused on their environmental occurrence, accumulation in various organisms, and preliminary toxicological effects, with ecotoxicological studies linking them to adverse impacts on aquatic species, including oxidative stress, reproductive harm, and disruptions to metabolic processes. However, critical knowledge gaps remain: existing literature has yet to fully elucidate the specific mechanisms of action of microplastics with varying physicochemical properties, the long-term effects of low-dose chronic exposure, and synergistic pathways with other environmental pollutants, which are inadequately explored.

Against this backdrop, this study aims to systematically review research on MPs/NPs and delve into the potential molecular mechanisms by which they contribute to CRC development. Our analysis will focus on human exposure pathways to these particles, their distribution and accumulation patterns in the body, the mechanisms underlying their ability to trigger or advance CRC, and strategies for assessing health risks and intervening in their impacts on colorectal health.

## 2 Research methods and retrieval strategy

To comprehensively collect literature relevant to this research topic, we systematically searched multiple authoritative online databases, including PubMed, Scopus, Google Scholar, Science Direct, ProQuest, EMBASE, Semantic Scholar, and Web of Science, spanning the period from 2004 to 2024 to encompass both historical and recent studies. Search terms included “microplastics or nanoplastics” “impact of microplastics or nanoplastics on human health” “adverse or harmful effects of microplastics or nanoplastics” “microplastics or nanoplastics induced cancer” “microplastics or nanoplastics induced oxidative stress” “microplastics or nanoplastics induced inflammation” “toxic effects or reactions of microplastics or nanoplastics” and “microplastics or nanoplastics and tumorigenesis.” An initial search identified 1,067 published research reports addressing the potential human health impacts of MPs and NPs. The study selection process strictly adhered to the PRISMA (Preferred Reporting Items for Systematic Reviews and Meta-Analyses) checklist (Supplementary Figure 2).

The inclusion criteria encompassed:

- Study designs: Original research articles including randomized controlled trials, cohort studies, epidemiological studies, cross-sectional analyses, and experimental studies (*in vivo* and *in vitro*)
- Preliminary reviews were screened for background information
- Focus: MPs/NPs exposure pathways, bioaccumulation, molecular mechanisms, and associations with colorectal carcinogenesis

Exclusion criteria were strictly applied as follows:

- Duplicate publications
- Non-English articles
- Studies unrelated to biological health impacts (e.g., materials science, environmental monitoring without health endpoints)
- Incomplete or inaccessible data (e.g., missing methodology, unreported outcomes)
- Retracted articles or publications with editorial notices

After rigorous screening, 87 highly relevant and reliable studies were selected for synthesis.

## 3 Exposure and absorption routes of MPs and NPs into the human body

**Figure 3:**
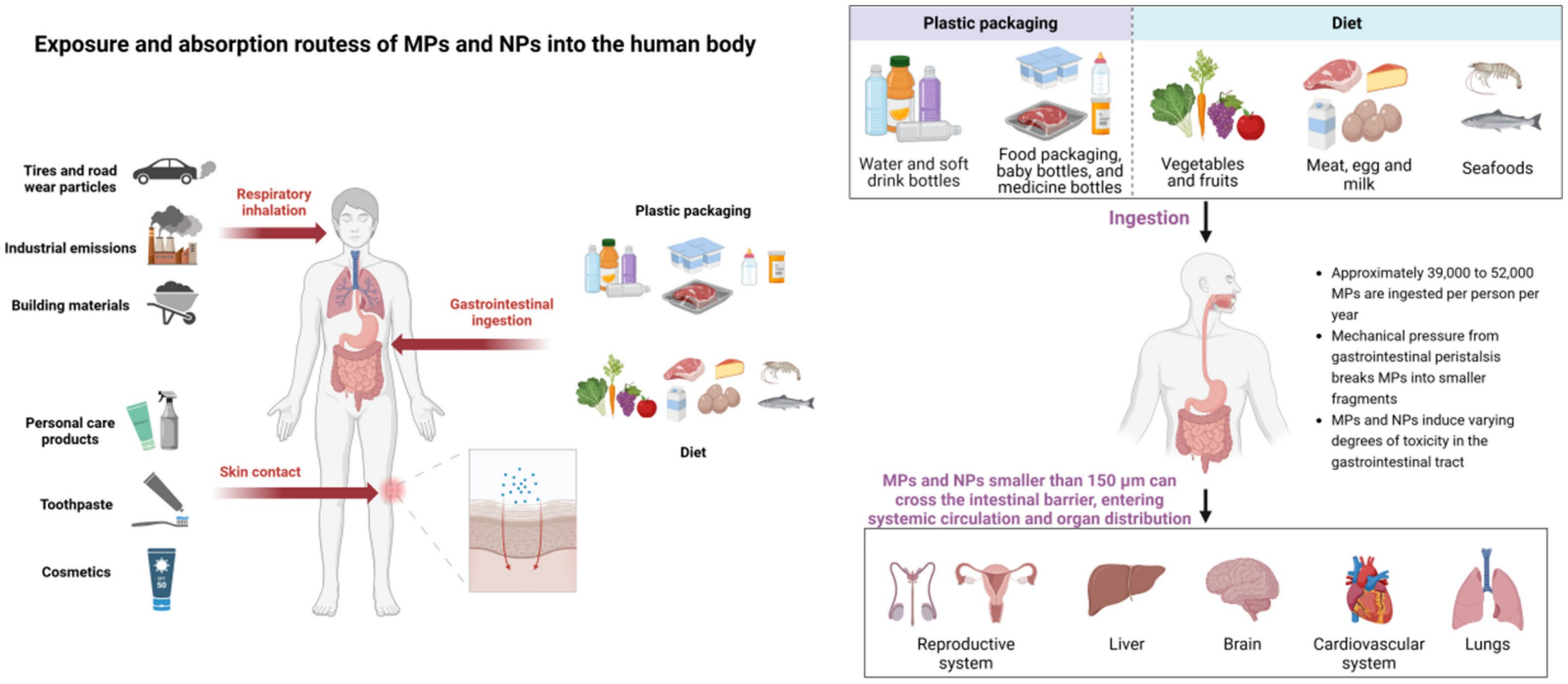
Exposure and absorption routes of MPs and NPs into the human body. Created with BioRender.com.

MPs/NPs spread globally through ocean currents, rivers, agricultural irrigation, and industrial wastewater, even entering atmospheric circulation via evaporation to reach remote areas like the Arctic (22). They pose food chain risks, with food packaging and containers creating additional exposure pathways. MPs/NPs in the environment can enter the human body through three main pathways: ingestion via the gastrointestinal tract, inhalation through respiration, and contact through the skin. Among these, ingestion and inhalation are the primary ways humans are exposed to MPs/NPs, while skin contact is a potential exposure route (Figure 3).

### 3.1 Gastrointestinal ingestion

The gastrointestinal tract is the primary entry route for MPs/NPs (23). People often unknowingly ingest MPs/NPs by consuming food or beverages contaminated with plastic particles. Various studies have reported the presence of MPs/NPs in table salt, seafood, vegetables, and fruits (24). Based on dietary habits, it is estimated that each American ingests between 39,000 and 52,000 MPs annually (13). Moreover, bottled water has been found to contain 325 to 10,000 MPs per liter (25).

In the gut, MPs/NPs exhibit low degradation without specific degrading enzymes (26), though digestive fluids may alter their surface properties, enhancing toxicity by promoting adhesion to intestinal walls and toxin absorption (27). Additionally, gastrointestinal peristalsis agitates MPs/NPs through mechanical pressure, causing fragmentation into smaller particles (28). Evidence for potential intestinal absorption of these particles comes from their detection in human feces, with an average of 20 MPs per 10 grams (29). Further support comes from *in vitro* studies, which show efficient uptake of nanoplastics by human intestinal cells (e.g., up to 14% uptake for those derived from hot beverage containers) (18). Animal studies reinforce this, revealing that 100 nm polystyrene particles have a 15-250-fold higher intestinal absorption rate (0.1–1%) compared to larger particles. In contrast, larger microplastics rarely penetrate the gut barrier, with absorption rates below 0.01% (19). Notably, studies indicate that MPs/NPs can inhibit efflux pumps in human colorectal adenocarcinoma cells and induce cytotoxicity. This raises heightened concerns about their potential role in the development and progression of CRC (30).

### 3.2 Respiratory inhalation

Inhalation of air and dust is another major exposure route for humans to MPs/NPs, with non-vigorous adult males inhaling up to 272 MPs daily (31). Sources include synthetic textiles, building materials, tire debris, and indoor dust, where 33% of settled dust comprises petrochemical fibers like PP (32, 33). These airborne particles can contaminate food, increasing gastrointestinal ingestion risk.

MPs/NPs concentration is higher in densely populated, industrial, and low-altitude areas (34). For humans, most particles in the upper respiratory tract are either expelled or swallowed; in the lungs, while phagocytosis and lymphatic transport clear most particles, some may still accumulate. Furthermore, *in vivo* studies have demonstrated that NPs can cross alveolar epithelial cells to enter the bloodstream (23). In the respiratory system, MPs/NPs’ large surface area causes dust overload, releasing chemokines, impairing macrophages, and inducing chronic inflammation, as shown by neutrophil accumulation and pro-inflammatory gene upregulation in rat lung cells exposed to PS-NPs (35).

### 3.3 Dermal contact

The skin serves as a vital physical barrier against external threats. However, exposure to personal care products (e.g., toothpaste, shampoo), cosmetics, or contaminated water droplets can enable MP/NP absorption (36). MPs, larger than skin pores, rarely penetrate the stratum corneum, whereas NPs can infiltrate through aged or damaged skin, sweat glands, or hair follicles (37). Once inside, NPs may induce oxidative stress in epithelial cells, posing health risks (35).

## 4 The journey of MPs/NPs in the human body: internalization and translocation

When MPs/NPs are ingested by the human body, if the body’s defense mechanisms cannot eliminate them, they may accumulate in cells or tissues or undergo translocation, which means that MPs/NPs penetrate beyond the surface of epithelial cells and embed deeper within cells or tissues. MPs and NPs exhibit remarkable morphological diversity, including fibers (e.g., microplastic fibers with circular or flattened cross-sections), spherical particles, irregular fragments, and films. Accumulating evidence underscores that this morphological variation plays a pivotal role in their biological behavior and toxicity, critically influencing their internalization and translocation pathways.

The translocation process is affected by several key factors, including the size of MPs/NPs, surface charge, and particle concentration, and crucially, particle shape. Shape-dependent differences in mobility directly influence bioavailability. For instance, flattened microplastic fibers have a settling velocity over 450% lower than cylindrical ones, enabling them to persist longer in the atmosphere and facilitating more efficient long-distance transport. Similarly, compared to volume-equivalent spherical particles, the slender geometry of fibers significantly reduces sedimentation in biological fluids, enhancing their potential to infiltrate diverse compartments (38). Smaller MPs/NPs generally translocate more efficiently than larger ones, as they can more easily cross cell membranes or paracellular pathways, while larger ones rely more on active transport (39). Cationic particles adhere more readily to cell surfaces, promoting active transport, and higher particle concentrations increase interaction likelihood (40).

**Figure 4:**
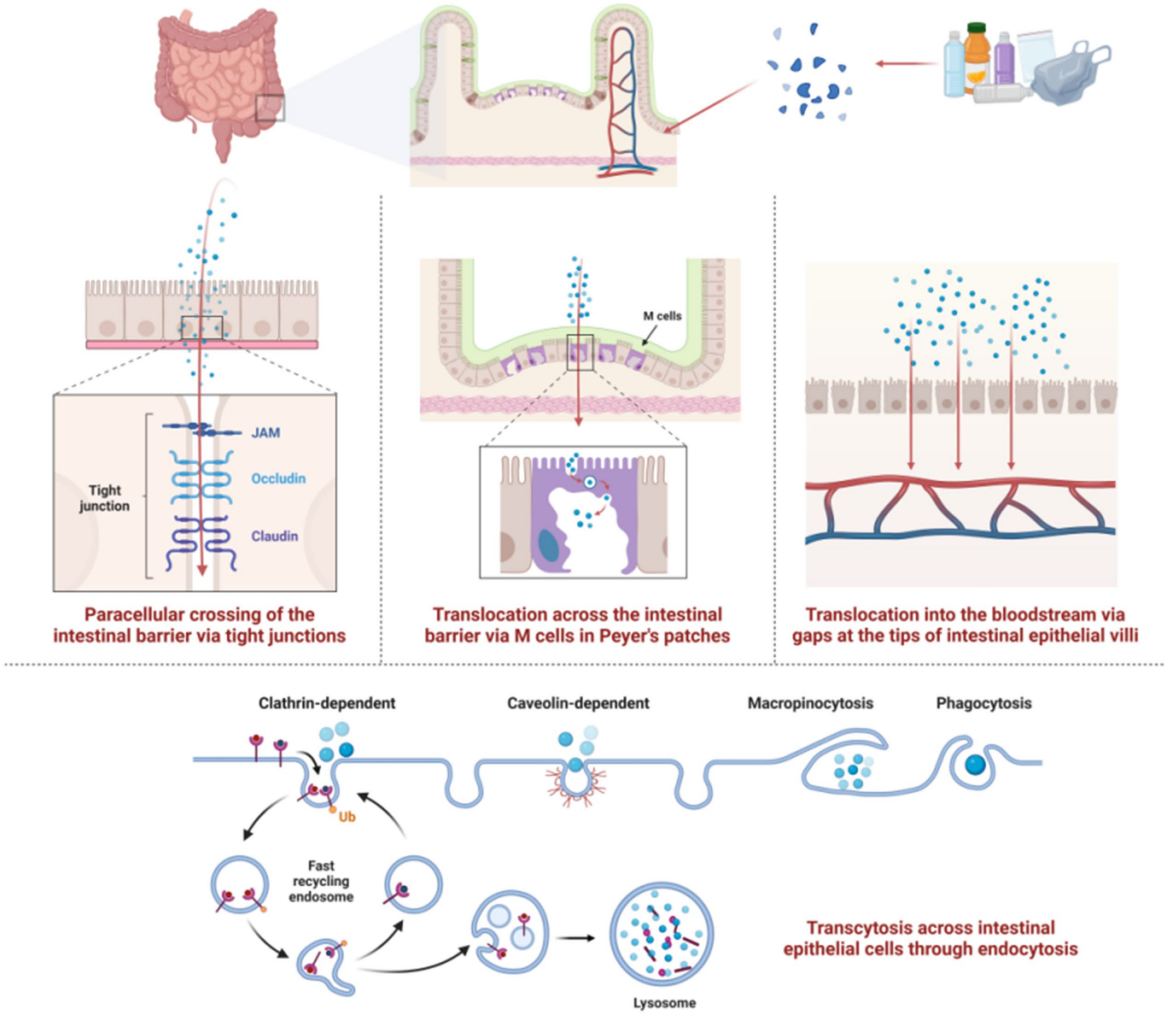
The four pathways by which MPs/NPs cross the intestinal barrier. Created with BioRender.com.

When the size of MPs exceeds 150 μm, they usually become encapsulated by intestinal mucus upon contact with the apical surface of intestinal epithelial cells, preventing further penetration of the intestinal wall (41). In contrast, MPs/NPs smaller than 150 μm can potentially cross the intestinal barrier and enter the lymphatic and blood circulation systems (4). Through research on the translocation mechanisms of MPs/NPs in the human body, four main pathways for intestinal barrier penetration have been identified (42) (Figure 4). Small-sized NPs can diffuse into the bloodstream through the paracellular pathways between tight junctions, aided by mucus secreted by goblet cells in the intestinal epithelium. Larger NPs (50–200 nm) tend to penetrate intestinal epithelial cells via endocytosis, a process where cells actively uptake external substances by forming vesicles through specialized membrane proteins. Intestinal cells use various endocytic pathways like macropinocytosis, phagocytosis, caveolin-dependent, and clathrin-dependent endocytosis. Microfold cells (M cells) in the Peyer’s patches of the intestine, a specialized type of intestinal epithelial cells in gut-associated lymphoid tissue, can uptake MPs/NPs by phagocytosis. Additionally, MPs can enter the bloodstream through the “gaps” formed by shedding or damaged intestinal cells at the tips of the villi.

Inhaled MPs/NPs deposit on respiratory mucosa and are mainly cleared by coughing and ciliary motion, but mechanical removal is inefficient due to their small size (43). In the alveoli, macrophages engulf MPs for lymphatic removal, yet microfibers (63% being MPs > 20 μm) are detected in 58% of cancer patients’ lung tissues (44). MPs/NPs (20–20,000 nm) in the digestive tract or trachea can translocate through epithelia into the lymphatic and circulatory systems, distributing to organs like the liver, spleen, and kidneys (45). Smaller particles have greater mobility. MPs with a size of 150 μm or less are absorbed by lymph nodes, and NPs with a size of 50 nm or less penetrate cell membranes to reach lysosomes, mitochondria, and the nucleus, which can alter organelle function (46). Inside cells, NPs act as “Trojan horses,” releasing toxins and pathogens to exacerbate cellular damage (47).

## 5 MPs/NPs as drivers in CRC: potential carcinogenic pathways and toxic effects

While direct studies linking MPs/NPs exposure to CRC incidence are lacking, existing research indicates that MPs/NPs may contribute to CRC onset and progression via pathways like ERK and TGF-*β* (48). Once in the gastrointestinal system, MPs/NPs can increase intestinal permeability, disrupt gut microbiota composition, and alter metabolic activity (49). Crucially, the physical and biological impact of MPs/NPs is significantly influenced by their morphology. Irregular fragments cause more severe physical damage through mechanical abrasion due to their angular surfaces. Fibrous MPs, with their elongated structure, promote prolonged contact with cellular membranes, triggering distinct biological responses such as oxidative stress, DNA damage, immune activation, and intestinal inflammation, elevating levels of inflammatory markers such as IL-6, IL-1*α*, and TNF-α (50). They also may promote angiogenesis (51). Additionally, MPs/NPs can reshape the colonic microbiota and reduce colonic mucosal thickness, potentially causing dysbiosis (52). NPs further exhibit shape-dependent toxicity. Surface topology, a shape-related property influenced by functional groups modifying surface charge (e.g., amine or carboxyl), critically modulates interactions with immune cells - PS-NH₂ inhibits macrophage phagocytosis, whereas PS-COOH does not - highlighting how shape-related surface properties intersect with chemistry to alter toxicity mechanisms (46, 47).

In summary, ingested MPs/NPs accumulate in the intestines, interacting with intestinal tissues through digestive peristalsis. These interactions may contribute to intestinal inflammation, microbiota disruption, and structural changes. MPs/NPs may also induce DNA damage, oxidative stress, and intestinal barrier dysfunction, all of which are associated with tumorigenesis (24). To fully understand the carcinogenic potential of MPs/NPs in CRC, further research into their mechanisms of action is needed. Systematic studies are required to clarify their roles in CRC development and inform prevention strategies.

### 5.1 MPs/NPs-induced DNA damage and genomic instability

MPs/NPs with a diameter of less than 50 nm have the ability to penetrate the cell nucleus, where they can deeply enter cells and cause DNA damage, even inducing apoptosis. The impact of MPs/NPs on DNA involves complex indirect mechanisms, mainly through inducing oxidative stress. Excessive reactive oxygen species (ROS) generated by MPs/NPs directly attack DNA. For instance, ROS can oxidize guanine in DNA to form 8-oxoguanine, leading to base mispairing, replication errors, and genetic mutations. ROS can also cleave the deoxyribose backbone of DNA, resulting in single-stranded or double-stranded breaks, with double-stranded breaks being one of the most severe forms of DNA damage. Additionally, ROS promotes the formation of covalent bonds between DNA and proteins, disrupting DNA replication and transcription processes and exacerbating genomic instability. Unrepaired DNA damage in intestinal epithelial cells can lead to gene dysfunction or oncogene activation, driving CRC development. Moreover, genomic instability increases tumor cell drug resistance, complicating CRC treatment (53, 54).

### 5.2 MPs/NPs-induced oxidative stress

The cytotoxicity of MPs/NPs is mainly characterized by the induction of oxidative stress through three pathways: surface-adsorbed oxidized substances boost ROS release while suppressing antioxidant enzymes; MPs/NPs trigger inflammatory responses that further increase ROS production; and metal oxides carried by MPs/NPs contribute to this process. When intracellular and extracellular oxidative substances overwhelm the cell’s antioxidant capacity, redox balance is disrupted, causing lipid peroxidation, protein inactivation, and DNA damage. ROS can directly damage genetic material and interfere with cell signaling, which is vital for cancer cell growth. Studies in zebrafish and mouse models show that MP exposure increases ROS and antioxidant enzyme levels (34). Nano-sized particles, due to their unique properties, are more potent in inducing antioxidant responses than microplastics (55). *In vitro* experiments reveal that NPs increase ROS levels in human colonic mucosal epithelial cells (56). Additionally, MPs/NPs with larger surface areas generate more ROS (57), intensifying oxidative stress.

### 5.3 MPs/NPs-induced intestinal inflammation

A balanced immune response is essential for pathogen elimination, yet an overactive response can damage tissues, with inflammation being a key indicator. Both animal and *in vitro* studies demonstrate that MPs/NPs, recognized as foreign by the immune system, trigger inflammatory responses upon accumulation (23). MPs/NPs induce inflammation through two main mechanisms: first, by causing oxidative stress that depletes antioxidant defenses (58), and second, by abrading epithelial cells in the intestine, which promotes inflammatory cell infiltration (24).

**Figure 5:**
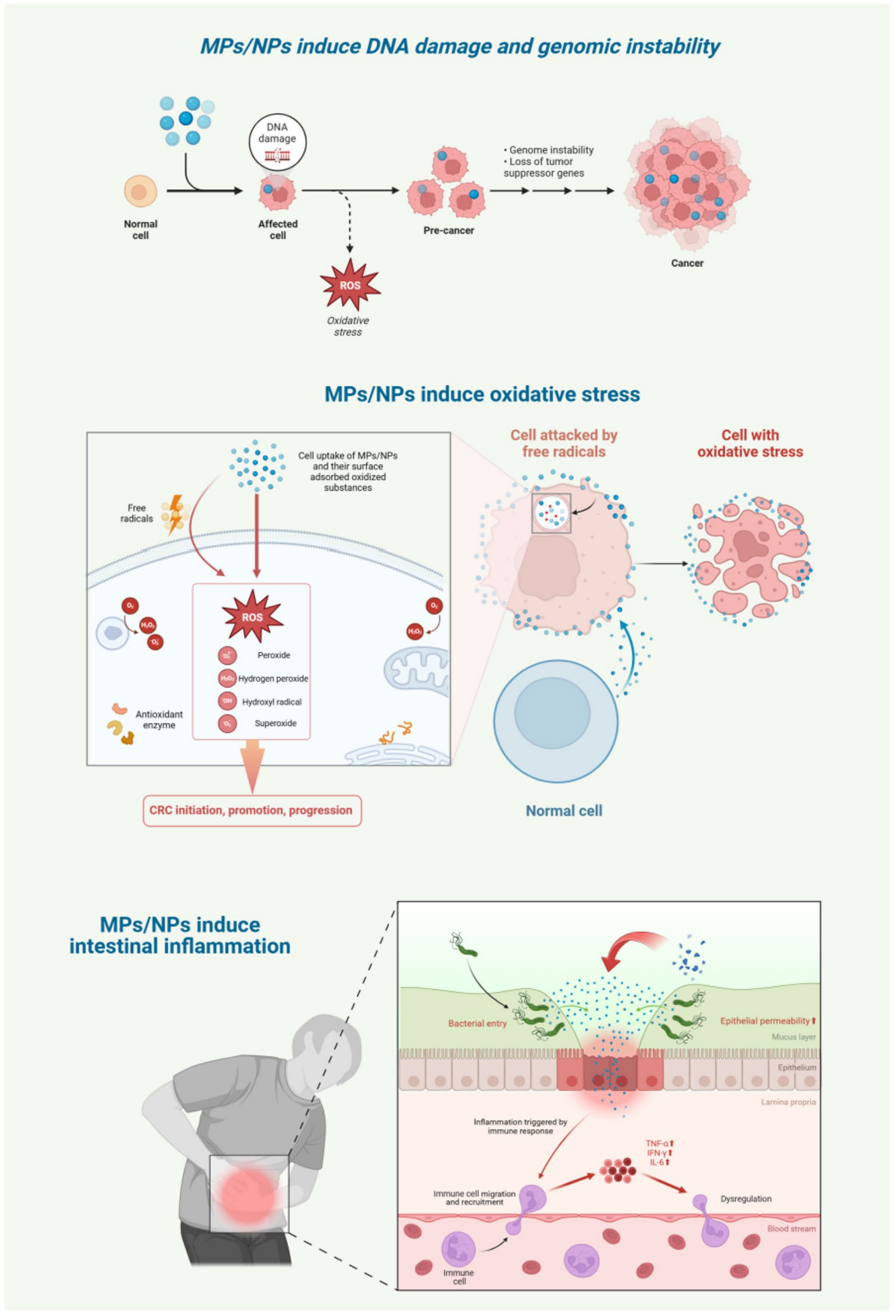
MPs/NPs induce DNA damage, genomic instability, oxidative stress and intestinal inflammation. Created with BioRender.com.

Experimental evidence supports these pathways. Mice ingesting polyethylene MPs show increased pro-inflammatory transcription factor expression and chronic inflammatory cell infiltration in the colon and duodenum lamina propria, confirming intestinal inflammation. Similarly, juvenile guppy fish exposed to PS-MPs for 28 days exhibit elevated levels of pro-inflammatory cytokines like TNF-*α*, IFN-*γ*, and IL-6 (59). These cytokines drive tumorigenesis by promoting cell proliferation, inhibiting apoptosis, and enhancing metastasis. They also act on tight junction proteins, inducing intestinal epithelial cell apoptosis and weakening the intestinal barrier, thus worsening inflammation (60). Normally, the intestinal epithelial barrier restricts MP/NP transport. However, in inflammatory bowel disease patients, increased intestinal permeability can more than double MP translocation across the intestinal mucosa (58). The chronic inflammation induced by MPs/NPs significantly heightens the risk of malignant transformation in vulnerable cells (Figure 5).

### 5.4 Carrier effect of MPs/NPs: Trojan horse for toxic compounds and opportunistic pathogens

MPs/NPs are characterized by their large surface area and strong adsorption capacity. Despite their small size, these particles can absorb and accumulate various toxic compounds, including antibiotics, heavy metals, additives, chemical pollutants, as well as opportunistic pathogens, facilitating the spread of these substances through water, soil, and air (44). A prime example is bisphenol A (BPA), which is widely used in plastic and resin production. BPA released from MPs/NPs can damage the intestinal barrier integrity, exacerbate intestinal inflammation, and increase the risk of colorectal cancer cell proliferation (48). Research shows that BPA causes overexpression in colorectal cancer cells by interfering with the estrogen - induced apoptosis cascade through estrogen receptor *β*, which is commonly present in the human intestine, thus weakening the protective effect of endogenous estrogen against colorectal cancer cell growth (61). The harmful effects of BPA on the human body have been well - studied (62). It can trigger local inflammation, affect colon cell permeability, and increase the levels of IFN-*γ*, IL-17, and immunoglobulin A, further disrupting immune function and microbiome balance. These changes can promote pro-tumor inflammation and accelerate the development of CRC. Additionally, organisms exposed to MPs/NPs have been found to contain higher levels of various environmental pollutants, such as polycyclic aromatic hydrocarbons, polybrominated diphenyl ethers, and polychlorinated biphenyls, compared to non-exposed individuals (58).

**Figure 6:**
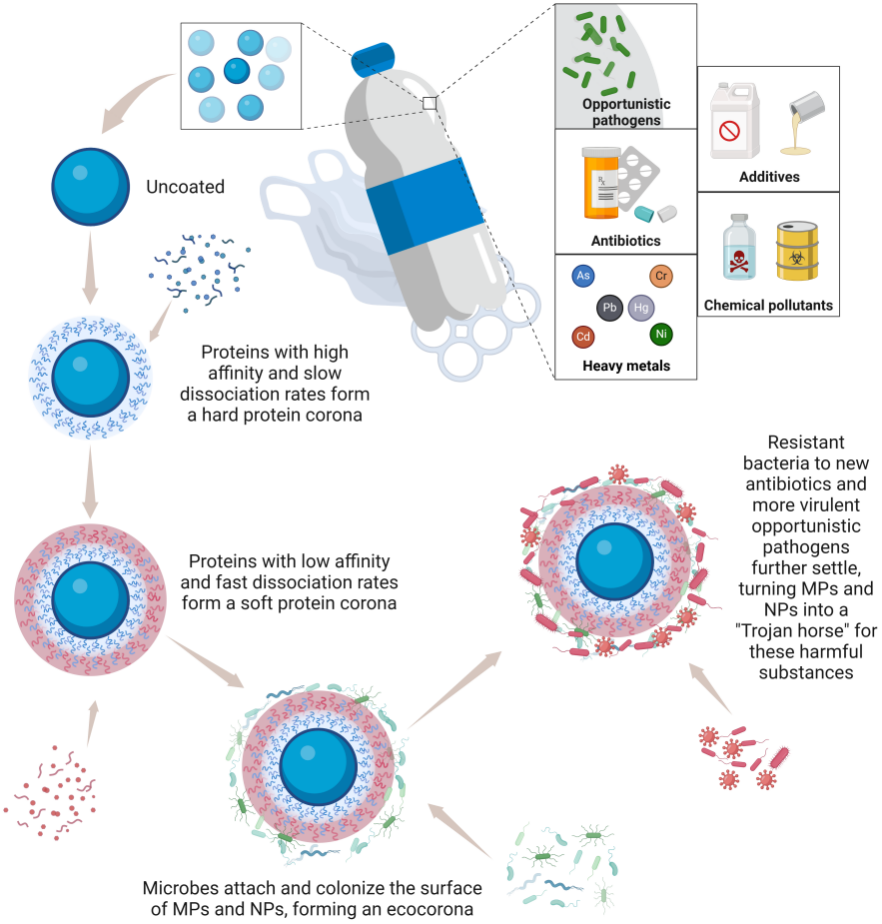
MPs and NPs act as a “Trojan horse” for toxic compounds and opportunistic pathogens. Created with BioRender.com.

When MPs/NPs enter the human body, they are coated with proteins, forming a “protein corona.” This nutrient-rich layer attracts gut microbiota, leading to the formation of an “eco corona” on the surface of MPs (63) (Figure 6). Studies have demonstrated that bacteria like *Escherichia coli* can bind to MPs and use them to deliver carcinogenic toxins to the colonic epithelium, thereby increasing the risk of colorectal cancer (30). This supports the idea that MPs can carry and transmit harmful bacteria in the colon, promoting carcinogenesis. Moreover, MPs/NPs not only serve as carriers for pathogenic microorganisms but may also influence bacterial evolution, promoting the emergence of new antibiotic-resistant and more virulent strains (64). The high concentration of antibiotics adsorbed on the surface of MPs/NPs can also facilitate the spread of resistance genes. For example, fluoroquinolone antibiotics (levofloxacin, ciprofloxacin) and penicillin antibiotics (amoxicillin) have been shown to adsorb onto PVC and polyamide MPs/NPs, confirming this phenomenon (14, 65).

### 5.5 MPs/NPs’ impact on intestinal barrier function

**Figure 7:**
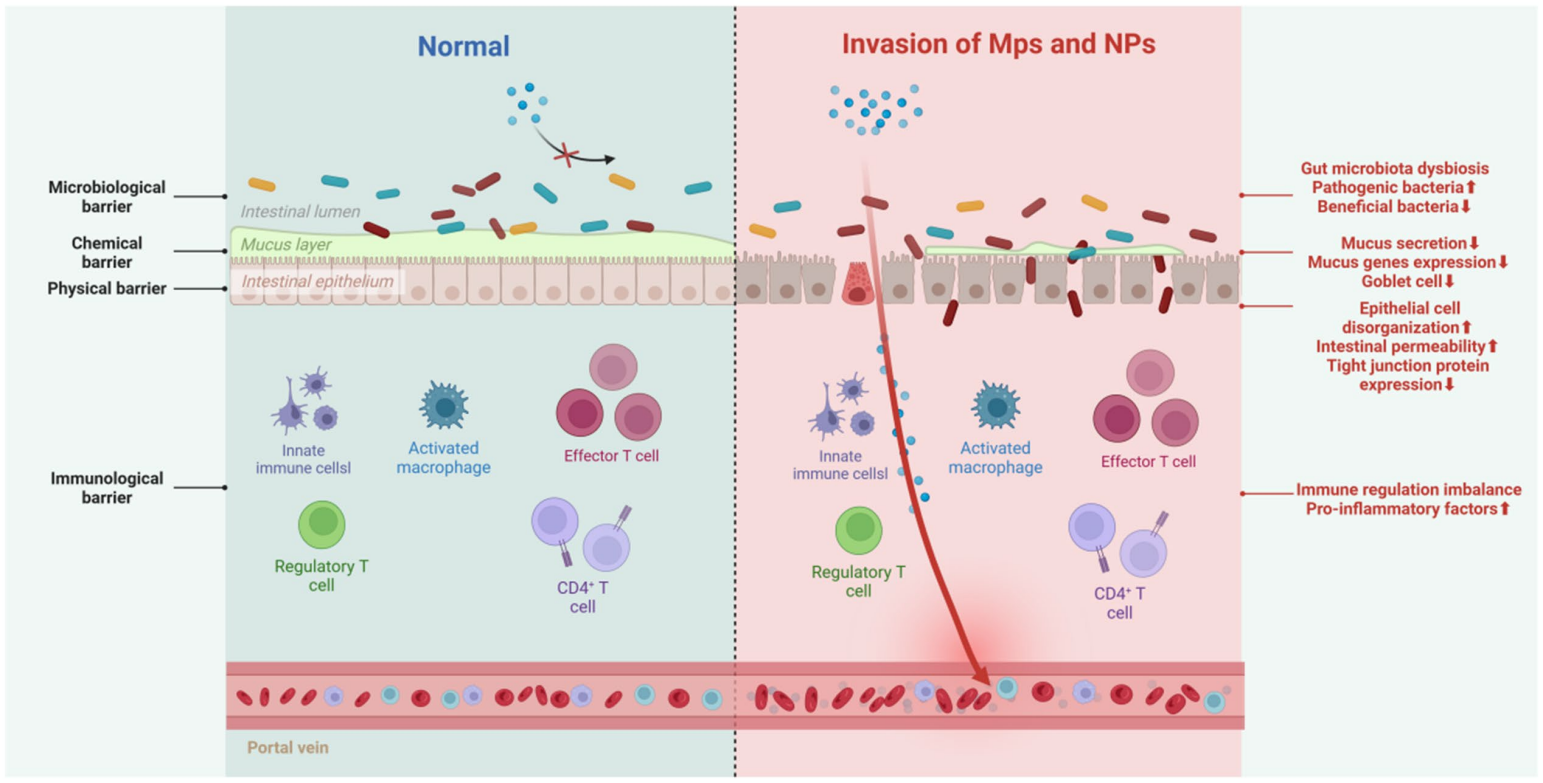
Impact of MPs/NPs on intestinal barrier function. Created with BioRender.com.

The intestines are vital for both nutrient absorption and barrier protection. The intestinal barrier, a sophisticated system formed by the interplay of intestinal epithelial cell junctions, secretions, immune cells, and gut microbiota, safeguards the body against bacteria, pathogens, and foreign particles. Comprising physical, chemical, biological, and immune components, these barriers collaborate to maintain internal environmental stability (66) (Figure 7).

#### 5.5.1 Physical barrier

The intestinal physical barrier, made up of tight junctions formed by protein complexes, stops intestinal contents from leaking and blocks bacteria, toxins, and inflammatory mediators in the intestinal lumen from entering the bloodstream. Exposure to MPs deforms intestinal epithelial cells in Artemia parthenogenetic larvae (67), increases intestinal permeability in zebrafish (68), and reduces the transcription of tight junction proteins Zo-1 and claudin-1 in the colon and ileum of mice (69). Decreased tight junction protein expression also occurs in avian models exposed to microplastics (70).

#### 5.5.2 Chemical barrier

The intestinal chemical barrier, primarily the mucus layer, comprises antimicrobial peptides, epithelial-secreted mucus, and microbiota-produced inhibitors. It inactivates pathogens, lubricates the mucosa, reduces MP-cell contact, and uses charge repulsion against MPs. Gastrointestinal mucus’s adhesiveness helps clear MPs (11, 71). However, MPs can undermine this barrier. In various species, including goldfish larvae (72), European sea bass (73), zebrafish (74), and mice (75), MP exposure causes intestinal mucosal damage, reduces goblet cell numbers, and thins the mucus layer. In mice, MP exposure also decreases mucin - related gene expression. A thinner mucus layer allows carcinogens to directly contact intestinal cells, increasing the risk of cancer. Additionally, the charge-repulsion mechanism may affect the absorption and diffusion of other substances, complicating intestinal health.

#### 5.5.3 Biological barrier

The biological barrier, mainly composed of the normal intestinal microbiota, resists the colonization of foreign bacteria. Disrupting this microbial balance can allow opportunistic and conditional pathogens to invade. In nature, MPs and microorganisms interact bidirectionally: some soil microorganisms can degrade MPs, and MPs can affect microbial communities (11). While research on whether the human intestinal microbiota can degrade MPs is limited, it is clear that MPs/NPs can interact directly with intestinal bacteria or act as carriers of antibiotics, selectively killing sensitive bacteria, promoting resistant bacteria growth, causing microbial imbalance, and triggering intestinal inflammation. For example, PS-MPs cause abnormal changes in the intestinal microbiota of peacock fish and zebrafish larvae, increasing the relative abundance of Proteobacteria and decreasing Actinobacteria (59, 76). In adult zebrafish, exposure to MPs reduces intestinal microbial diversity (4). In mice, PS-MPs decrease mucin secretion, leading to a significant reduction in the relative abundance of Firmicutes and *α*-Proteobacteria (77).

#### 5.5.4 Immunological barrier

The immunological barrier consists of gut-associated lymphoid tissue and various immune cells, which coordinate immune responses by presenting antigens, producing antibodies, and secreting cytokines. Macrophages and lymphocytes are the primary targets of the immunotoxicity of MPs/NPs. When PS-NPs enter human THP-1 macrophages, they induce an increase in ROS, leading to nuclear damage and a decrease in mitochondrial membrane potential, thereby reducing cell viability (78). Upon exposing adult zebrafish to 500 μg/L of PS-MPs for 21 days, a decrease in the proportion of M1 macrophages and altered chemotaxis of B cells were observed (79). Additionally, exposure to polyethylene reduced the percentage of Th17 and Treg cells in mice, leading to immune dysregulation (80). Research by HU et al. has indicated that MPs exposure may induce reproductive toxicity by disrupting immune homeostasis (81). In mice with intestinal immune imbalance exposed to PS-MPs, levels of pro-inflammatory factors such as TNF-*α*, IL-1β, and IFN-*γ* increased, while the expression of the antioxidant activity gene GPx was upregulated, severely disrupting the colonic microbiota and metabolism (82). Although research in this area is still insufficient, there is substantial evidence suggesting that MPs possess immunotoxicity *in vivo*. This indicates that plastic-induced damage may affect immune cells, including those within the gut immune system.

## 6 Discussion and future directions

MPs/NPs are abundant, widespread, and resistant to degradation, mainly entering the body through food and water ingestion. Their carcinogenic potential is concerning, as once they cross tissue barriers, they can circulate, releasing harmful substances and triggering oxidative stress, cytotoxicity, and genotoxicity, contributing to CRC. Research on MPs/NPs has shifted from environmental studies to human health impacts, but understanding their carcinogenic mechanisms, especially in CRC, is still limited. Three key knowledge gaps need addressing:

- Comprehensive carcinogenic risk assessment: to fully evaluate the carcinogenic risk of MPs/NPs, it is crucial to elucidate their health effect endpoints, identify sensitive evaluation indicators, and establish dose–response relationships, revealing the mechanisms and pathways of their toxicological effects. Previous studies have often been based on high-concentration, short-term exposure conditions; however, in real-world scenarios, MPs/NPs exposure levels are typically lower, but their bioaccumulation and degradation over time can influence their carcinogenic risk. Therefore, future research should focus on low-concentration and lifecycle-based assessments of MPs/NPs toxicity in humans. This can be achieved through collaboration among medical researchers, ecologists, and epidemiologists to construct population cohorts exposed to environmental MPs/NPs and corresponding bioaccumulation models across different geographical regions. By considering various exposure pathways, sources, quantities, as well as factors like geography, age, gender, occupation, and lifestyle, the long-term carcinogenic potential of MPs/NPs under chronic exposure can be explored.
- Differences in toxic responses: the toxic responses of MPs/NPs in animals and humans differ, and existing cell and organ models cannot fully replicate the complexities of the human body. Therefore, caution is needed when extrapolating these experimental results to assess the risk of MPs/NPs to human health. Once ingested, MPs/NPs may undergo digestion, bind with lipids and nucleic acids, and experience changes in their physical and chemical properties (such as particle size and adsorption characteristics), affecting their toxicity. Future research should further investigate how changes in the intrinsic properties of microplastics after entering the body alter their toxicological mechanisms.
- Variability in experimental conditions: there are discrepancies between the materials, particle sizes, concentrations, and morphologies of MPs/NPs used in experiments and those found in the actual environment. This mismatch fails to account for the complexity of MPs/NPs in real-world settings, including variations in size, shape, polymer composition, surface morphology, and degree of weathering. Smaller particle sizes and higher concentrations can enhance the accumulation and toxicity of MPs/NPs, which necessitates focused study. Particularly, the role of MPs/NPs as carriers of harmful substances and the potential interactive and combined toxic effects with other environmental pollutants and harmful microorganisms or pathogens require close attention.

In conclusion, MPs/NPs, as global pollutants, pose a complex and urgent challenge to human health. Given their potential to induce various carcinogenic mechanisms, which may interact with each other, there is a pressing need for more comprehensive research from multiple angles and levels. Such research can provide scientific evidence for developing targeted prevention and control strategies and help us better understand the potential threats of MPs/NPs to human health. We strongly advocate for interdisciplinary collaboration to promote a thorough understanding and study of the health impacts of MPs/NPs, laying the groundwork for constructing a more robust protective system.

## 7 Conclusion

This systematic review comprehensively synthesizes two decades of research to elucidate the potential carcinogenic roles of MPs and NPs in CRC development, filling critical knowledge gaps in understanding their mechanistic links to carcinogenesis. Key findings reveal that MPs/NPs, via ingestion, inhalation, and dermal contact, translocate across biological barriers to induce DNA damage, oxidative stress, and intestinal barrier disruption by reducing tight junction proteins, while triggering chronic inflammation through pro-inflammatory cytokines and gut microbiota dysbiosis. Their unique role as “Trojan horses”—adsorbing toxicants like bisphenol A and pathogens—further exacerbates cytotoxicity and activates carcinogenic pathways, highlighting a multifaceted contribution to CRC initiation and progression. This review innovatively integrates insights into how MPs/NPs with varying physicochemical properties (size, polymer type) mediate these effects, addressing previously underexplored aspects such as long-term low-dose exposure impacts and synergistic interactions with other pollutants. By clarifying these mechanisms, the work not only underscores the significant carcinogenic risk of MPs/NPs in CRC but also provides a foundational framework for scientific management of MPs/NPs pollution, emphasizing the need for future research on lifecycle-based toxicity assessments, species-specific toxic responses, and the complex interplay between MPs/NPs and environmental co-contaminants to inform targeted prevention and control strategies.

## Data Availability

The original contributions presented in the study are included in the article/Supplementary material, further inquiries can be directed to the corresponding authors.
