# Supplementary figures and images for: Invisible invaders: unveiling the carcinogenic threat of microplastics and nanoplastics in colorectal cancer-a systematic review
Source: Front Public Health. 2025 Aug 19;13:1653245. doi: 10.3389/fpubh.2025.1653245 (PMC12402728; doi:10.3389/fpubh.2025.1653245)

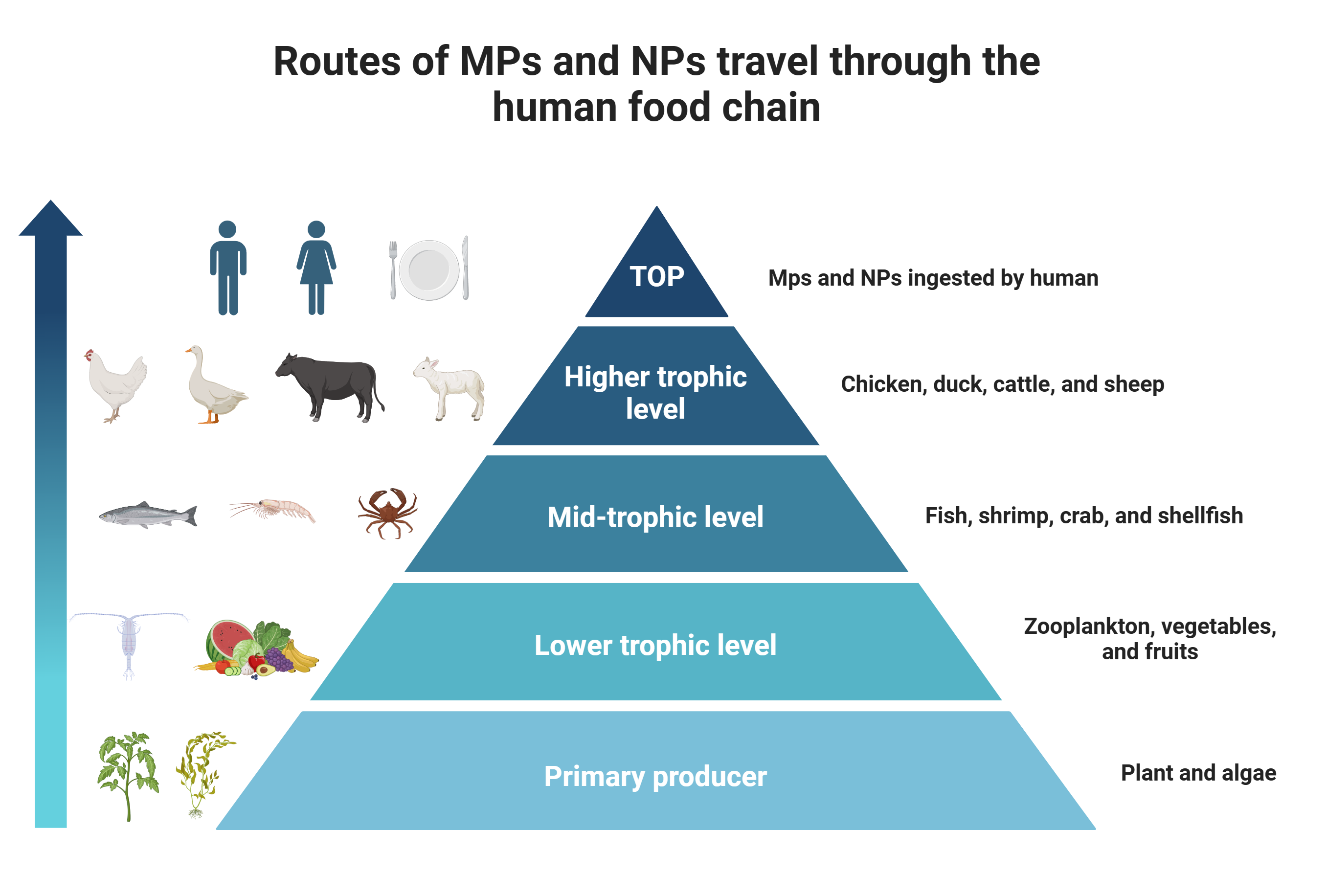

Supplement: Supplementary file 1 [file Image_1.png]

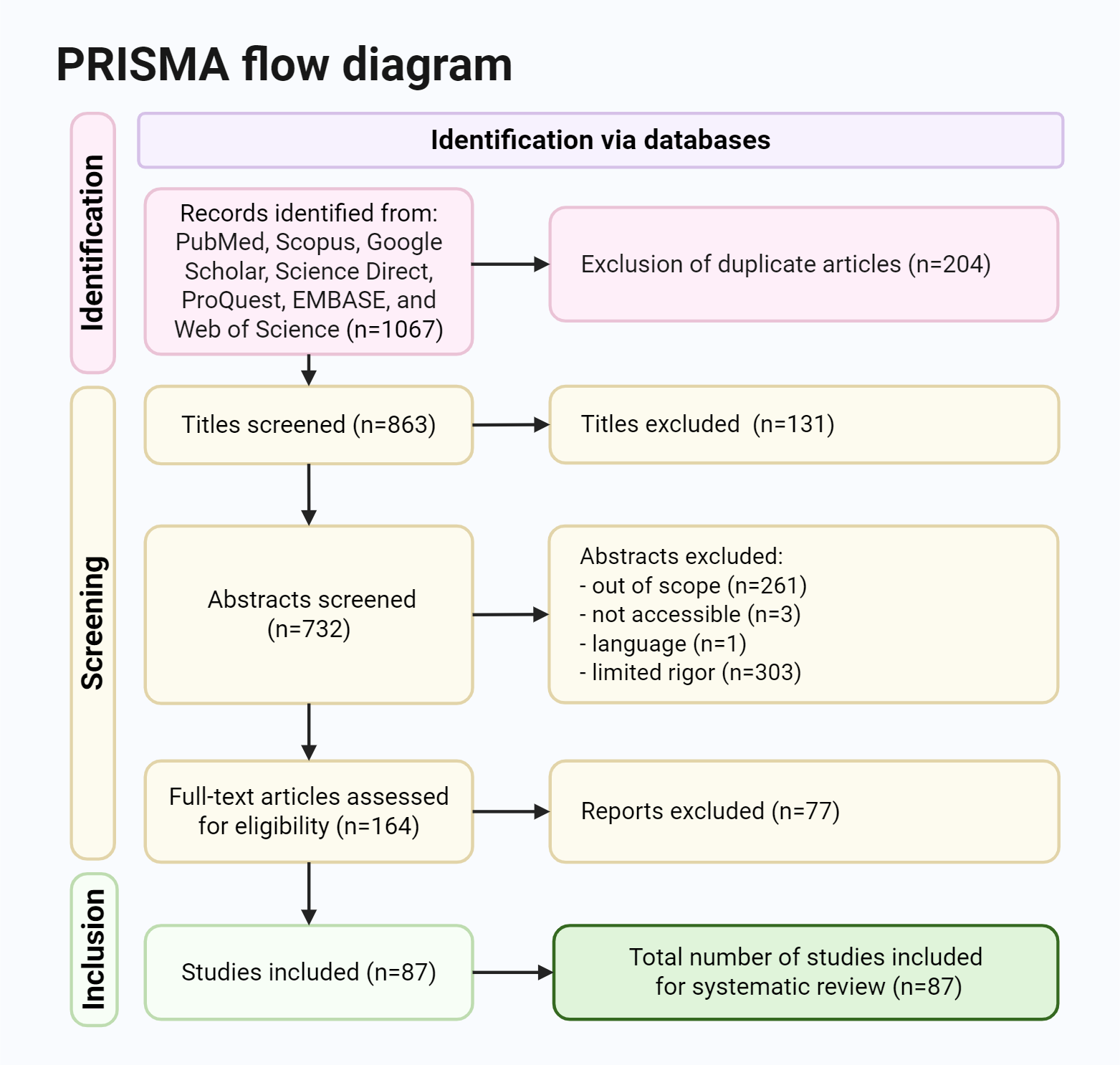

Supplement: Supplementary file 2 [file Image_2.png]

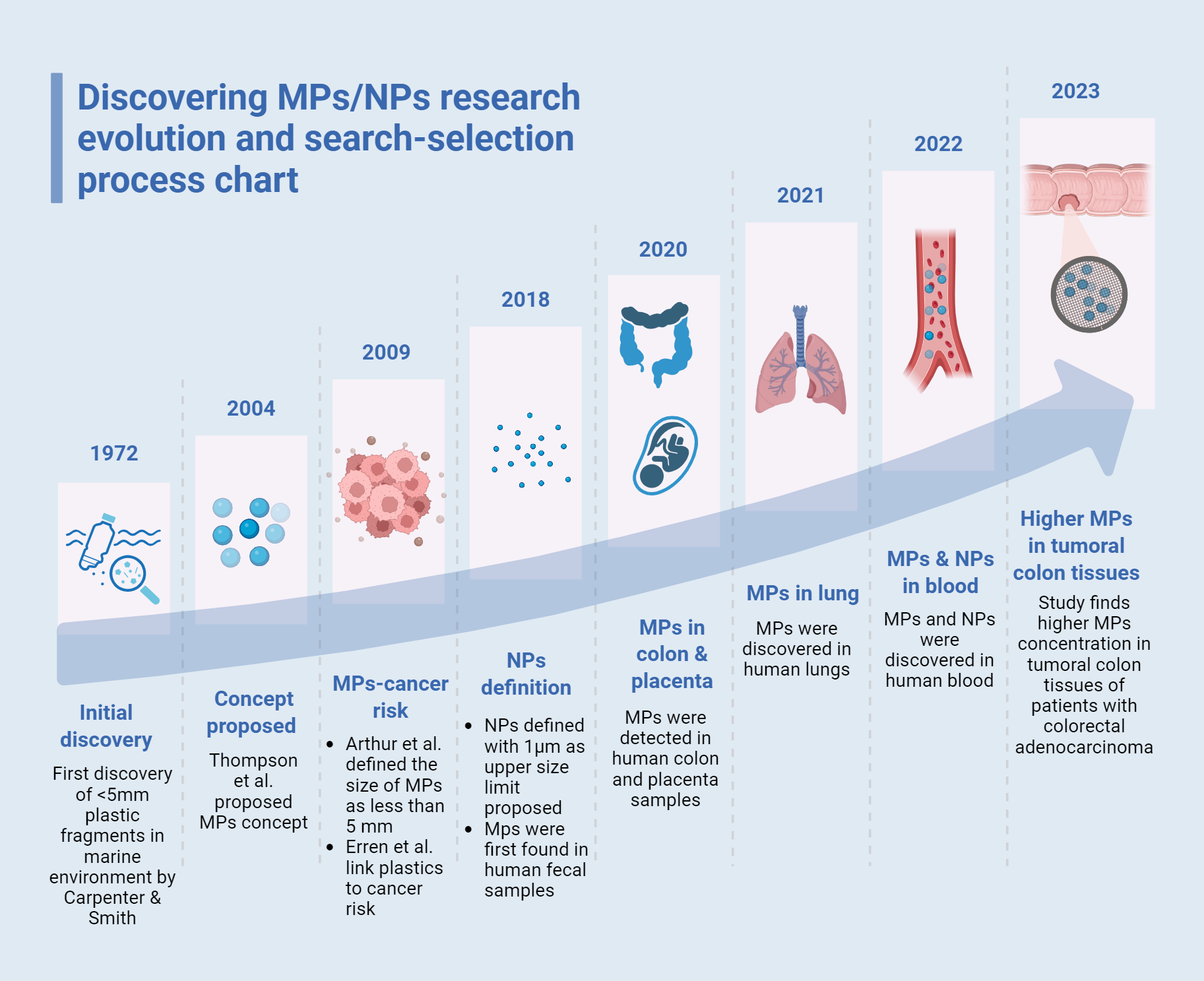

Supplement: Supplementary file 3 [file Image_3.png]
